# Time-dependent damage in predictions of fatigue behaviour of normal and healing ligaments

**DOI:** 10.1007/s11043-015-9267-7

**Published:** 2015-06-10

**Authors:** Gail M. Thornton, Soraya J. Bailey, Timothy D. Schwab

**Affiliations:** 1 McCaig Institute for Bone and Joint Health, Departments of Surgery and Civil Engineering, University of Calgary, 3A18 Health Research Innovation Centre, 3280 Hospital Drive NW, Calgary, Alberta Canada T2N 4Z6; 2 Department of Orthopaedics, University of British Columbia, Vancouver, British Columbia Canada; 3 School of Health Sciences, University of Northern British Columbia, Prince George, British Columbia Canada

**Keywords:** Creep, Fatigue, Damage, Ligament, Healing

## Abstract

Ligaments are dense fibrous tissues that connect bones across a joint and are exposed daily to creep and fatigue loading. Ligaments are tensile load-bearing tissues; therefore, fatigue loading will have a component of time-dependent damage from the non-zero mean stress and cycle-dependent damage from the oscillating stress. If time-dependent damage is not sufficient to completely predict the fatigue response, then cycle-dependent damage could be an important contributor. Using data from normal ligaments (current study and Thornton et al., Clin. Biomech. 22:932–940, 2007a) and healing ligaments (Thornton and Bailey, J. Biomech. Eng. 135:091004-1–091004-6, 2013), creep data was used to predict the fatigue response considering time-dependent damage. Relationships between creep lifetime and test stress or initial strain were modelled using exponential or power-law regression. In order to predict fatigue lifetimes, constant rates of damage were assumed and time-varying stresses were introduced into the expressions for time-dependent damage from creep. Then, the predictions of fatigue lifetime were compared with curvefits to the fatigue data where exponential or power-law regressions were used to determine the relationship between fatigue lifetime and test stress or initial strain. The fatigue prediction based on time-dependent damage alone greatly overestimated fatigue lifetime suggesting that time-dependent damage alone cannot account for all of the damage accumulated during fatigue and that cycle-dependent damage has an important role. At lower stress and strain, time-dependent damage was a greater relative contributor for normal ligaments than healing ligaments; however, cycle-dependent damage was a greater relative contributor with incremental increases in stress or strain for normal ligaments than healing ligaments.

## Introduction

Ligaments are biological soft tissues that connect bones across a joint. During normal daily activities, ligaments are subjected to cyclic and static loads (Holden et al. 1994), thereby experiencing fatigue and creep. The majority of the wet weight of ligament is water and the majority of the dry weight is collagen, predominantly type I collagen. The major tensile load-bearing component of ligament is collagen, which is arranged in a hierarchical fashion with collagen crosslinks (Frank et al. 1995), bimodal distribution of collagen fibril diameters (Frank et al. 1992) and crimp patterns that relate to collagen fibre recruitment (Thornton et al. 2002). When a ligament is healing from an acute injury, it undergoes three overlapping stages (Frank et al. 1999): inflammation, matrix production and matrix remodelling. Even after the matrix remodelling stage, some features of healing ligaments may never return to normal values; for example, decreased ultimate tensile strength (UTS) (Chimich et al. 1991), reduced collagen crosslinks (Frank et al. 1995), more unimodal distribution of fibril diameters (Frank et al. 1992), more glycosaminoglycans that bind water (Frank et al. 1983) and increased number of flaws in the matrix (Shrive et al. 1995) have been observed at the 14 week healing interval following surgical injury to the medial collateral ligament (MCL) in the rabbit model (Thornton et al. 2000). Given that ligaments are exposed to creep and fatigue and that healing ligaments have decreased UTS compared to normal ligaments, differences may exist in how damage is accumulated comparing normal and healing ligaments where decreased UTS may increase the susceptibility of healing ligaments to damage. The relative importance of time-dependent and cycle-dependent damage may have clinical implications when designing rehabilitation exercises, which involve sustained and repeated loading, following injury to a complementary ligament restraint of the joint or to the ligament itself.

Rabbits are a common model for studying ligament and tendon mechanics, with a variety of breeds being studied: Japanese White (Yamamoto and Hayashi 1998), New Zealand White (Woo et al. 1992; Danto and Woo 1993; Thornton et al. 2002), and Burgunder (Schwab et al., 2007; Thornton et al., 2007a, 2007b). The rabbit MCL is often used to study normal and healing ligament mechanical properties. While we had previously reported the similarity of the UTS of MCLs from two of these breeds: New Zealand White $95.4 \pm 12.3~\mbox{MPa}$ ($n=15$) and Burgunder $97.7 \pm12.6~\mbox{MPa}$ ($n=5$) (Thornton et al. 2007a), we have yet to compare their creep and fatigue behaviour. Our first purpose in this study was to evaluate the creep and fatigue behaviour of MCLs at 60 %UTS from two rabbit breeds to determine if data collected previously for skeletally-mature, female Burgunder rabbits (Thornton et al. 2007a) is comparable to that for skeletally-mature, female New Zealand White rabbits.

If the creep and fatigue data from normal MCLs from the two rabbit breeds were similar, then data collected previously for creep and fatigue behaviour of healing MCLs from skeletally-mature female New Zealand White rabbits (Thornton and Bailey 2012; Thornton and Bailey 2013) could be compared to the creep and fatigue data from the normal MCLs from the two rabbit breeds. Our second purpose was to compare the creep and fatigue response of normal and healing ligament extracellular matrix (ECM) during long-term loading in vitro. For normal ligaments, we have previously shown that the fatigue lifetime is shorter than the creep lifetime at test stresses ranging from 15 % to 60 % of the UTS of normal ligaments (Thornton et al. 2007a). For healing ligaments, we have previously shown that fatigue lifetime is shorter than creep lifetime at test stresses ranging from 30 % to 80 % of the UTS of healing ligaments (Thornton and Bailey 2013). Because of the decreased UTS of the healing ligaments compared to the normal ligaments, only one test stress was comparable, which was the lowest for the normal ligaments and the highest for the healing ligaments, and the lifetimes for healing ligaments were at least four orders of magnitude shorter than the lifetimes of normal ligaments. What remains to be investigated is whether the relative contribution of time-dependent and cycle-dependent damage to the fatigue response of the ECM of normal and healing ligaments is different. Tensile fatigue loading is an oscillating stress about a non-zero mean stress, which means that fatigue loading will have a component of time-dependent damage from the non-zero mean stress and cycle-dependent damage from the oscillating stress. If a prediction of the fatigue response based on time-dependent damage alone formulated from creep data is insufficient to completely describe the actual fatigue response, then cycle-dependent damage could be an important contributor to the fatigue response.

## Methods

### Comparison of normal MCLs from different breeds

Five female one-year-old New Zealand White rabbits were used in this study approved by the University of Calgary Animal Care Committee. One MCL from each rabbit was assigned to creep and the other MCL to fatigue. The creep and fatigue tests for the normal MCLs were performed at 60 %UTS of MCLs from New Zealand White rabbits where UTS was $95.4 \pm 12.3~\mbox{MPa}$ ($n=15$) (Thornton et al. 2002). Testing was performed as described previously (Thornton and Bailey 2012; Thornton and Bailey 2013). After standardized preparation and mounting in an MTS system, the knee joint underwent two cycles from $-5~\mbox{N}$ to $+2~\mbox{N}$, stopping at $+1~\mbox{N}$ to establish ligament zero. After measuring MCL length with digital calipers and MCL midsubstance cross-sectional area with area calipers (Shrive et al. 1988), an environment chamber was equilibrated at 37°C and 99 % relative humidity before re-establishing ligament zero. The MCL underwent preconditioning for 30 cycles at 1 Hz from $+1~\mbox{N}$ to a force corresponding to 4.8 MPa (5 % of 95.4 MPa). Fatigue-tested MCLs underwent cyclic loading using a 1 Hz sine wave from $+1~\mbox{N}$ to a force corresponding to 57.2 MPa (60 % of 95.4 MPa). Creep-tested MCLs experienced sustained loading at the same test stress as in the fatigue tests, but the creep test was occasionally interrupted with an unloading/reloading cycle to monitor modulus (Thornton et al. 2007b; Thornton and Bailey 2012). Lifetime was measured relative to the beginning of the initial loading to the test stress and was the last time the MCL reached 99 % of the desired test force. Strain was calculated as the deformation divided by the undeformed MCL length and initial strain was the strain when the MCL was initially loaded to the test stress. Creep and fatigue parameters were compared using Wilcoxon signed-rank tests for paired data. The MCL creep and fatigue data from New Zealand White rabbits were compared to previously published data for Burgunder rabbits (Thornton et al. 2007a) using Mann–Whitney $U$ tests.

### Time-dependent models

The experiments were performed for normal MCLs as detailed in the previous section of the current study and in Thornton et al. (2007a) and for healing MCLs as detailed in Thornton and Bailey (2013). Briefly, after failure tests to determine the UTS of normal and healing ligaments, creep and fatigue tests were performed at test stresses that were a percentage of the UTS of the normal and healing ligaments. Due to the decreased UTS of healing ligaments, only one test stress was comparable between normal and healing ligaments. During fatigue tests, the MCL was cycled from $+1~\mbox{N}$ to a force corresponding to the desired test stress using a sine wave at 1 Hz. During creep tests, the MCL was loaded from $+1~\mbox{N}$ to a force corresponding to the desired test stress using a sine wave at 1 Hz, and constant force was maintained except when interrupted occasionally with an unloading/reloading cycle using a sine wave at 1 Hz in order to monitor modulus (Thornton et al. 2007b; Thornton and Bailey 2012).

Creep and fatigue tests were performed at a variety of stresses for normal and healing ligaments. The UTS for normal ligaments from Burgunder rabbits was $97.7 \pm 12.6~\mbox{MPa}$ ($n=5$) (Thornton et al. 2007a), for normal ligaments from New Zealand White rabbits was $95.4 \pm12.3~\mbox{MPa}$ ($n=15$) (Thornton et al. 2002) and for healing ligaments from New Zealand White rabbits was $18.0 \pm 5.2~\mbox{MPa}$ ($n=7$) (Thornton and Bailey 2013). Normal ligaments were creep and fatigue tested at 60 %UTS ($n=10$) (current study) and 60 %UTS ($n=9$), 30 %UTS ($n=8$) and 15 %UTS ($n=5$) (Thornton et al. 2007a). Healing ligaments were creep and fatigue tested at 80 %UTS ($n=3$), 60 %UTS ($n=20$), 45 %UTS ($n=8$) and 30 %UTS ($n=9$) (Thornton and Bailey 2013). The test stresses for 30 %UTS to 60 %UTS of healing ligaments corresponded to 6 %UTS to 11 %UTS of normal ligaments, which are considered functional stresses. The test stress for 80 %UTS of healing ligaments was 15 %UTS of normal ligaments, which is at the transition from the toe region to linear region of the normal MCL stress–strain curve. The 30 %UTS to 60 %UTS of normal ligaments represented levels experienced by a normal ligament with injury to a complementary ligament restraint and were similar %UTS to the healing ligaments. Fatigue or creep lifetime was defined as either the last time the MCL reached 99 % of the desired test force, or the time when the deformation safety limit was reached, or the allocated test time of 24 hours or 24 hour intervals thereafter for a creep test, which exceeded the time of the longest fatigue test at the same test stress.

Relationships between creep lifetime and test stress, and creep lifetime and initial strain were modelled using exponential regression or power-law regression. In order to predict fatigue lifetimes, constant rates of damage were assumed, and time-varying stresses were introduced into the expressions for time-dependent damage from creep (Wren et al. 2003; Thornton and Bailey 2013). The relative importance of time-dependent damage and cycle-dependent damage was evaluated in two ways. First, the fatigue lifetime represented by the curvefit to the fatigue data was expressed as a percentage of the fatigue lifetime predicted from time-dependent damage when evaluated at similar %UTS, stress and strain for normal and healing ligaments. Second, the percent difference in the slope comparing the prediction from time-dependent damage to the curvefit to the fatigue data was calculated and compared for normal and healing ligaments.

#### Test stress

The relationship between creep lifetime and test stress was modeled using the exponential regression

1 $$ T_{\mathrm{life},\mathrm{creep},\mathrm{data}} = Ae^{ - B\sigma_{o}}, $$

where $T_{\mathrm{life},\mathrm{creep},\mathrm{data}}$ was the creep lifetime, $A$ and $B$ were the constants determined from regression, and $\sigma_{o}$ was the test stress.

Assuming a constant rate of damage, the creep damage was

2 $$ D ( t ) = \int_{0}^{t} \frac{1}{Ae^{ - B\sigma_{o}}}\,dt, $$

and the creep lifetime was determined at $D=1$.

A time-varying stress was introduced into Eq. (2) to predict the fatigue lifetime

3 $$ 1 = \int_{0}^{t} \frac{1}{Ae^{ - B [ ( 1/2 )\sigma_{\max} ( 1 + \sin2\pi ft ) ]}} \,dt, $$

where $\sigma_{\max}$ was the maximum cycle stress, $f$ was the frequency, and $t$ was the time (s). The fatigue lifetime ($T_{\mathrm{life},\mathrm{fatigue},\mathrm{predict}}$) was determined using the trapezoidal rule (trapz Matlab command).

In order to compare the fatigue prediction from Eq. (3) to a fatigue curvefit, the fatigue lifetime and test stress were modeled using the exponential regression

4 $$ T_{\mathrm{life},\mathrm{fatigue},\mathrm{data}} = Ae^{ - B\sigma_{o}}, $$

where $T_{\mathrm{life},\mathrm{fatigue},\mathrm{data}}$ was the fatigue lifetime, $A$ and $B$ were the constants determined from regression, and $\sigma_{o}$ was the test stress.

Rather than using exponential regression, a power-law regression could be used to relate the creep lifetime and test stress:

5 $$ T_{\mathrm{life},\mathrm{creep},\mathrm{data}} = U\sigma_{o}^{ - V}, $$

where $T_{\mathrm{life},\mathrm{creep},\mathrm{data}}$ was the creep lifetime, $U$ and $V$ were the constants determined from regression, and $\sigma_{o}$ was the test stress.

Assuming a constant rate of damage, the creep damage was

6 $$ D ( t ) = \int_{0}^{t} \frac{1}{U\sigma_{o}^{ - V}}\,dt, $$

and the creep lifetime was determined at $D=1$.

A time-varying stress was introduced into Eq. (6) to predict the fatigue lifetime

7 $$ 1 = \int_{0}^{t} \frac{1}{U [ ( 1/2 )\sigma_{\max} ( 1 + \sin2\pi ft ) ]^{ - V}} dt, $$

where $\sigma_{\max}$ was the maximum cycle stress, $f$ was the frequency, and $t$ was the time (s). The fatigue lifetime ($T_{\mathrm{life},\mathrm{fatigue},\mathrm{predict}}$) was determined using the trapezoidal rule (trapz Matlab command).

In order to compare the fatigue prediction from Eq. (7) to a fatigue curvefit, the fatigue lifetime and test stress were modeled using the power-law regression

8 $$ T_{\mathrm{life},\mathrm{fatigue},\mathrm{data}} = U\sigma_{o}^{ - V}, $$

where $T_{\mathrm{life},\mathrm{fatigue},\mathrm{data}}$ was the fatigue lifetime, $U$ and $V$ were the constants determined from regression, and $\sigma_{o}$ was the test stress.

#### Initial strain

The relationship between creep lifetime and initial strain was modeled using the exponential regression

9 $$ T_{\mathrm{life},\mathrm{creep},\mathrm{data}} = Ae^{ - B\varepsilon_{i}} $$

where $T_{\mathrm{life},\mathrm{creep},\mathrm{data}}$ was the creep lifetime, $A$ and $B$ were the constants determined from regression, and $\varepsilon _{i}$ was the initial strain.

Assuming a constant rate of damage, the creep damage was

10 $$ D ( t ) = \int_{0}^{t} \frac{1}{Ae^{ - B\varepsilon_{i}}} \,dt, $$

and the creep lifetime was determined at $D=1$.

To predict the fatigue lifetime, a time-varying stress was introduced into Eq. (10) by relating the initial strain to the test stress using the initial modulus

11 $$ 1 = \int_{0}^{t} \frac{1}{Ae^{ - B [ ( 1/2 )\varepsilon_{i} ( 1 + \sin2\pi ft ) ]}} \,dt, $$

where $f$ was the frequency, and $t$ was the time (s). The fatigue lifetime ($T_{\mathrm{life},\mathrm{fatigue},\mathrm{predict}}$) was determined using the trapezoidal rule (trapz Matlab command).

In order to compare the fatigue prediction from Eq. (11) to a fatigue curvefit, the fatigue lifetime and initial strain were modeled using the exponential regression

12 $$ T_{\mathrm{life},\mathrm{fatigue},\mathrm{data}} = Ae^{ - B\varepsilon_{i}}, $$

where $T_{\mathrm{life},\mathrm{fatigue},\mathrm{data}}$ was the fatigue lifetime, $A$ and $B$ were the constants determined from regression, and $\sigma_{o}$ was the test stress.

Rather than exponential regression, a power-law regression could be used to relate the creep lifetime and initial strain:

13 $$ T_{\mathrm{life},\mathrm{creep},\mathrm{data}} = U\varepsilon_{i}^{ - V}, $$

where $T_{\mathrm{life},\mathrm{creep},\mathrm{data}}$ was the creep lifetime, $U$ and $V$ were the constants determined from regression, and $\varepsilon _{i}$ was the initial strain.

Assuming a constant rate of damage, the creep damage was

14 $$ D ( t ) = \int_{0}^{t} \frac{1}{U\varepsilon_{i}^{ - V}}\,dt, $$

and the creep lifetime was determined at $D=1$.

To predict the fatigue lifetime, a time-varying stress was introduced into Eq. (14) by relating the initial strain to the test stress using the initial modulus

15 $$ 1 = \int_{0}^{t} \frac{1}{U [ ( 1/2 )\varepsilon_{i} ( 1 + \sin2\pi ft ) ]^{ - V}} \,dt, $$

where $f$ was the frequency, and $t$ was the time (s). The fatigue lifetime ($T_{\mathrm{life},\mathrm{fatigue},\mathrm{predict}}$) was determined using the trapezoidal rule (trapz Matlab command).

In order to compare the fatigue prediction from Eq. (15) to a fatigue curvefit, the fatigue lifetime and initial strain were modeled using the power-law regression

16 $$ T_{\mathrm{life},\mathrm{fatigue},\mathrm{data}} = U\varepsilon_{i}^{ - V}, $$

where $T_{\mathrm{life},\mathrm{fatigue},\mathrm{data}}$ was the fatigue lifetime, $U$ and $V$ were the constants determined from regression, and $\varepsilon _{i}$ was the initial strain.

## Results

### Comparison of normal MCLs from different breeds

At 60 %UTS, the creep lifetime was greater than the fatigue lifetime for normal MCLs from New Zealand White rabbits ($p= 0.04$; Table 1). Whereas the MCL length of MCLs from Burgunder rabbits was less than that of MCLs from New Zealand White rabbits ($p \leq 0.05$), the initial strain at 60 %UTS was not statistically different for the two breeds (Table 1). The creep lifetime was not different comparing MCLs from the two breeds when tested at the same percentage of UTS (Table 1). Likewise, the fatigue lifetime was not different when comparing MCLs from the two breeds (Table 1).

**Table 1:** Creep and fatigue of normal MCLs tested at 60 %UTS for New Zealand White rabbits and Burgunder rabbits

Test and Parameter	New Zealand White 60 %$\mathrm{UTS}=57.2~\mbox{MPa}$ Test Stress	Burgunder 60 %$\mathrm{UTS} = 58.6~\mbox{MPa}$ Test Stress
**Creep**		
Lifetime (s)	22.4 × 10^3^ (2.72 × 10^3^–62.6 × 10^3^)^∧^	21.2 × 10^3^ (10.2 × 10^3^–40.8 × 10^3^)^∧^
MCL Length (mm)	24.17 (23.01–26.40)^*^	21.70 (20.80–23.50)
Initial Strain (mm/mm)	0.090 (0.082–0.116)	0.104 (0.080–0.132)
*n*	5	4
**Fatigue**		
Lifetime (s)	2.46 × 10^3^ (0.296 × 10^3^–11.5 × 10^3^)	1.83 × 10^3^ (0.316 × 10^3^–3.65 × 10^3^)
MCL Length (mm)	25.27 (23.83–26.42)^*^	21.35 (20.05–22.05)
Initial Strain (mm/mm)	0.070 (0.069–0.118)	0.094 (0.089–0.111)
*n*	5	5

Data are shown as median (range)

New Zealand White rabbit normal MCL data is from the current study and Burgunder rabbit normal MCL data is from Thornton et al. (2007a)

^∧^Creep different than fatigue for same breed (*p* ≤ 0.04)

^*^New Zealand White different than Burgunder for same parameter (*p* ≤ 0.05)

### Time-dependent models

All fatigue test lifetimes were the last time the MCL reached 99 % of the desired test force except for one test when it was the time when the deformation safety limit was reached. The deformation safety limit was a safety limit for the MTS system during creep and fatigue testing, which was set to a value greater than the largest deformation during failure testing. Most creep test lifetimes were the last time the MCL reached 99 % of the desired test force except for eight tests when it was the allocated test time of 24 hours or 24 hour intervals thereafter for a creep test, which exceeded the time of the longest fatigue test at the same test stress.

**Fig. 1:**
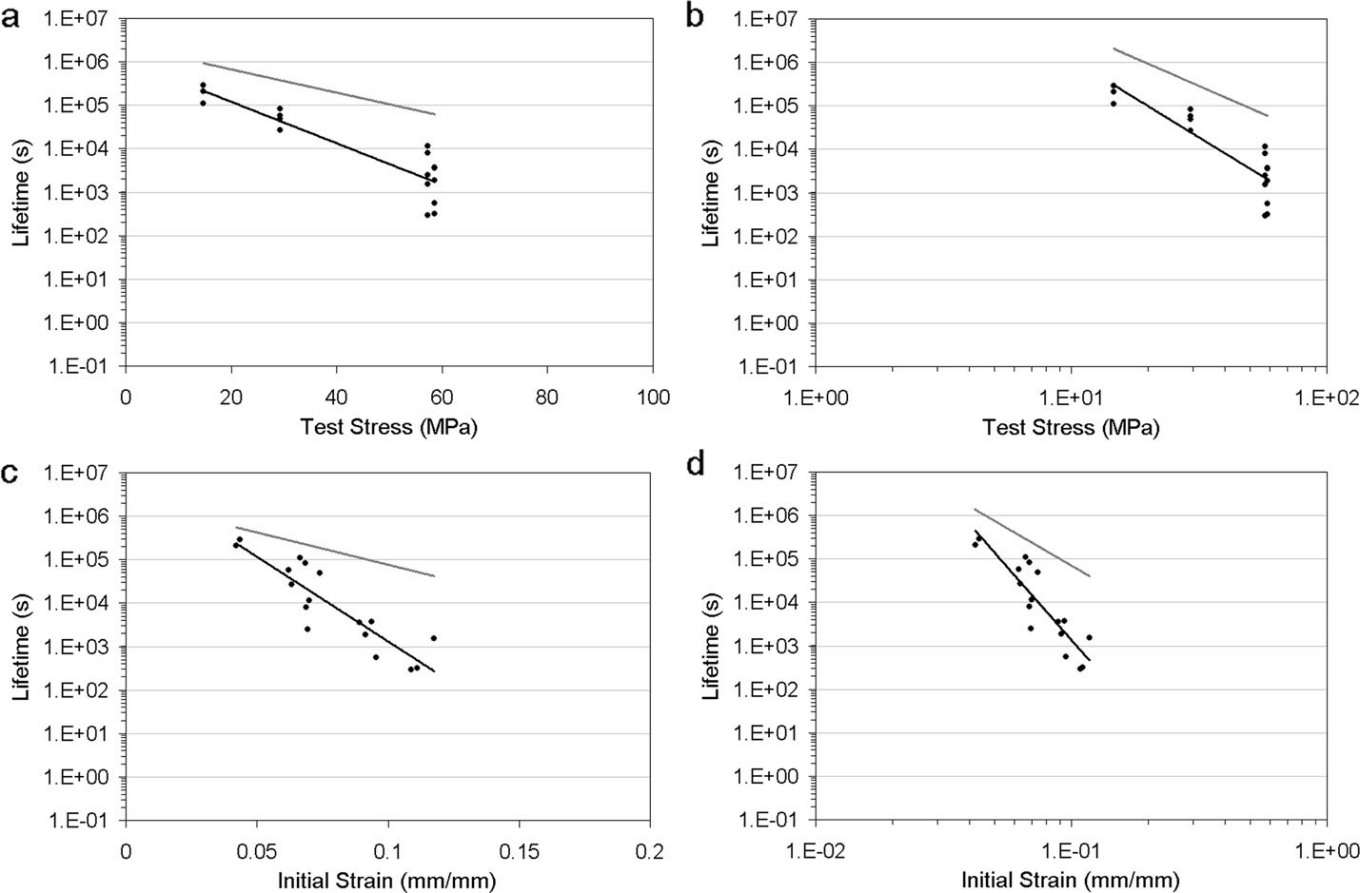
Normal ligament fatigue behaviour, showing data (*black circle*), curvefit from regression (*black line*; $T_{\mathrm{life},\mathrm{fatigue},\mathrm{data}}$), and prediction from time-dependent damage (*grey line*; $T_{\mathrm{life},\mathrm{fatigue},\mathrm{predict}}$). Normal MCL data is from the current study and Thornton et al. (2007a). (**a**) Test stress and exponential regression $T_{\mathrm{life},\mathrm{fatigue},\mathrm{data}} = 1.08 \times10^{6}e^{ - 0.110\sigma_{o}}$, $r^{2} =0.81$ and $T_{\mathrm{life},\mathrm{fatigue},\mathrm{predict}} = 2.29 \times10^{6}e^{ - 0.0616\sigma_{o}}$. (**b**) Test stress and power-law regression $T_{\mathrm{life},\mathrm{fatigue},\mathrm{data}} = 4.95 \times10^{9}\sigma_{o}^{ - 3.61}$, $r^{2} = 0.77$ and $T_{\mathrm{life},\mathrm{fatigue},\mathrm{predict}} = 2.01 \times 10^{9}\sigma_{o}^{ - 2.56}$. (**c**) Initial strain and exponential regression $T_{\mathrm{life},\mathrm{fatigue},\mathrm{data}} = 10.3 \times10^{6}e^{ - 89.7\varepsilon_{i}}$, $r^{2} = 0.79$ and $T_{\mathrm{life},\mathrm{fatigue},\mathrm{predict}} = 2.33 \times10^{6}e^{ - 34.1\varepsilon_{i}}$. (**d**) Initial strain and power-law regression $T_{\mathrm{life},\mathrm{fatigue},\mathrm{data}} = 280 \times10^{ - 6}\varepsilon_{i}^{ - 6.69}$, $r^{2} = 0.78$ and $T_{\mathrm{life},\mathrm{fatigue},\mathrm{predict}} = 26.5\varepsilon_{i}^{ - 3.42}$

**Fig. 2:**
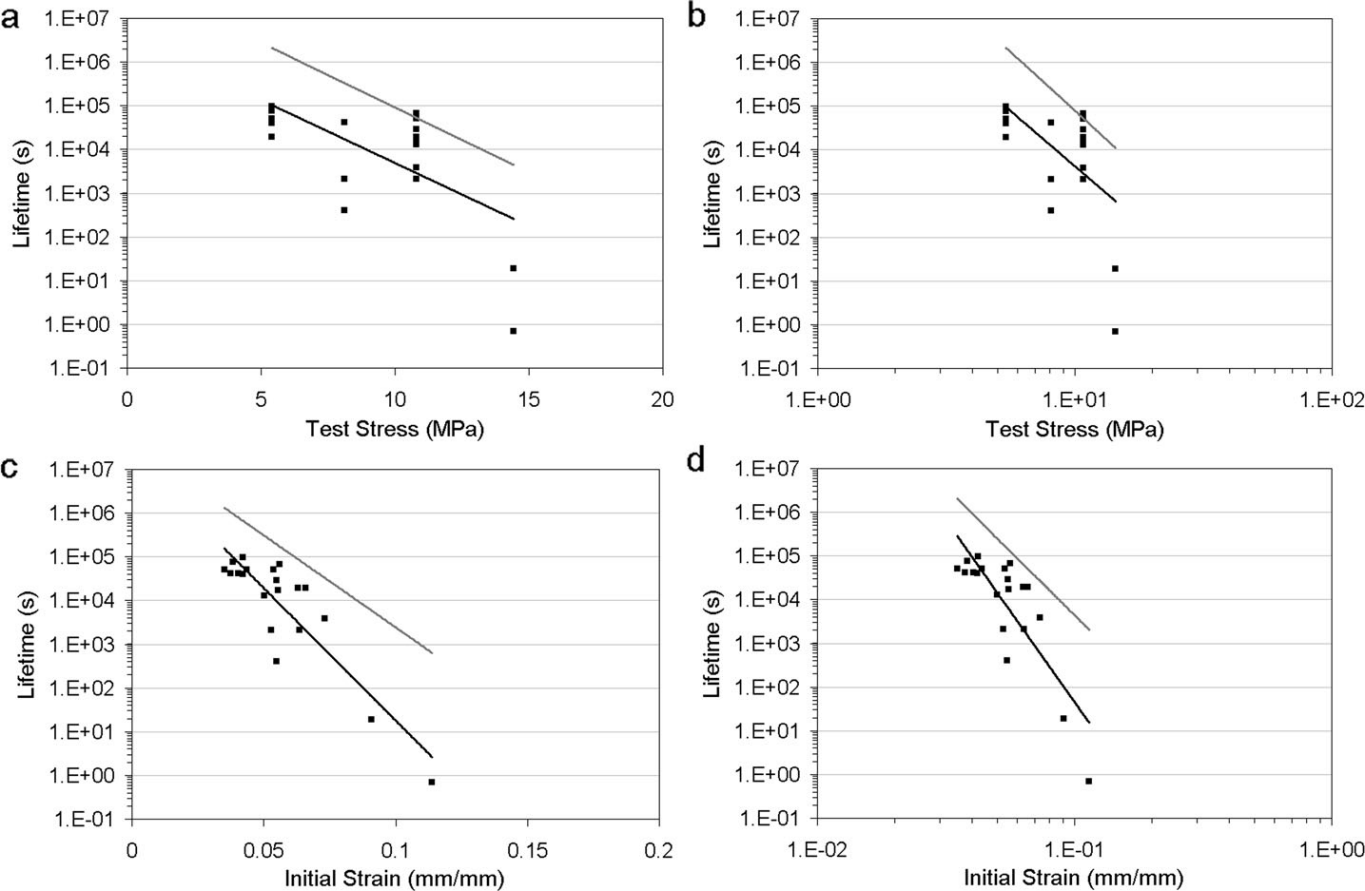
Healing ligament fatigue behaviour, showing data (*black square*), curvefit from regression (*black line*; $T_{\mathrm{life},\mathrm{fatigue},\mathrm{data}}$), and prediction from time dependent damage (*grey line*; $T_{\mathrm{life},\mathrm{fatigue},\mathrm{predict}}$). Healing MCL data is from Thornton and Bailey (2013). (**a**) Test stress and exponential regression $T_{\mathrm{life},\mathrm{fatigue},\mathrm{data}} = 3.78 \times10^{6}e^{ - 0.665\sigma_{o}}$, $r^{2} = 0.39$ and $T_{\mathrm{life},\mathrm{fatigue},\mathrm{predict}} = 84.1 \times10^{6}e^{ - 0.683\sigma_{o}}$. (**b**) Test stress and power-law regression $T_{\mathrm{life},\mathrm{fatigue},\mathrm{data}} = 480 \times10^{6}\sigma_{o}^{ - 5.05}$, $r^{2} = 0.30$ and $T_{\mathrm{life},\mathrm{fatigue},\mathrm{predict}} = 19.8 \times 10^{9}\sigma_{o}^{ - 5.40}$. (**c**) Initial strain and exponential regression $T_{\mathrm{life},\mathrm{fatigue},\mathrm{data}} = 20.5 \times10^{6}e^{ - 139\varepsilon _{i}}$, $r^{2} = 0.78$ and $T_{\mathrm{life},\mathrm{fatigue},\mathrm{predict}} = 39.7 \times10^{6}e^{ - 96.9\varepsilon_{i}}$. (**d**) Initial strain and power-law regression $T_{\mathrm{life},\mathrm{fatigue},\mathrm{data}} = 214 \times10^{ - 9}\varepsilon_{i}^{ - 8.33}$, $r^{2} = 0.68$ and $T_{\mathrm{life},\mathrm{fatigue},\mathrm{predict}} = 6.16 \times10^{ - 3}\varepsilon_{i}^{ - 5.86}$

The curvefits to the fatigue and creep data demonstrated that the fatigue lifetimes ($T_{\mathrm{life},\mathrm{fatigue},\mathrm{data}}$) were shorter than the creep lifetimes ($T_{\mathrm{life},\mathrm{creep},\mathrm{data}}$) for both normal and healing ligaments (Table 2, Figs. 1–2). For normal ligaments, the highest coefficient of determination to the creep data was achieved using a test stress model with exponential regression (Table 2). For normal ligament fatigue data, the coefficients of determination were around $r^{2} = 0.8$ for all models (Fig. 1). For healing ligaments, the highest coefficients of determination to the creep data and fatigue data were achieved using initial strain models with exponential regression (Table 2, Fig. 2).

**Table 2:** Exponential regressions for creep lifetime and test stress $T_{\mathrm{life},\mathrm{creep},\mathrm{data}} = Ae^{ - B\sigma_{o}}$ (1) and creep lifetime and initial strain $T_{\mathrm{life}.\mathrm{creep},\mathrm{data}} = Ae^{ - B\varepsilon_{i}}$ (9) and power-law regressions for creep lifetime and test stress $T_{\mathrm{life},\mathrm{creep},\mathrm{data}} = U\sigma_{o}^{ - V}$ (5) and creep lifetime and initial strain $T_{\mathrm{life},\mathrm{creep},\mathrm{data}} = U\varepsilon_{i}^{ - V}$ (13)

Model and Ligament	$T_{\mathrm{life},\mathrm{creep},\mathrm{data}}$ Exponential Regression			$T_{\mathrm{life},\mathrm{creep},\mathrm{data}}$ Power-Law Regression		
	*A* (s)	−*B*	$r^{2}$	*U* (s)	−*V*	$r^{2}$
**Test Stress**						
Normal Ligament	1.69 × 10^6^	−0.0782	0.74	658 × 10^6^	−2.56	0.66
Healing Ligament	33.9 × 10^6^	−0.741	0.36	4.64 × 10^9^	−5.39	0.27
**Initial Strain**						
Normal Ligament	1.53 × 10^6^	−41.8	0.50	8.17	−3.41	0.57
Healing Ligament	16.1 × 10^6^	−105	0.62	1.49 × 10^−3^	−5.84	0.47

#### Test stress

For the test stress models, the fatigue lifetime ($T_{\mathrm{life},\mathrm{fatigue},\mathrm{data}}$) was less than the predicted fatigue lifetime ($T_{\mathrm{life},\mathrm{fatigue},\mathrm{predict}}$) derived from the time-dependent damage from creep for both normal and healing ligaments. Evaluating the normal and healing ligaments at similar %UTS (30 %UTS–60 %UTS) where the fatigue lifetime represented by the curvefit to the data ($T_{\mathrm{life},\mathrm{fatigue},\mathrm{data}}$) was expressed as a percentage of the predicted fatigue lifetime ($T_{\mathrm{life},\mathrm{fatigue},\mathrm{predict}}$) revealed similar values for normal and healing ligaments (Table 3). When compared at the one comparable stress (${\sim}14~\mbox{MPa}$) that was the lowest for the normal ligaments (15 %UTS) and the highest for the healing ligaments (80 %UTS), the fatigue lifetime was a greater percentage of the predicted lifetime for normal ligaments compared with healing ligaments (Table 3). Using exponential regression, the percent difference in slope (constant $-B$) comparing the fatigue prediction from creep to the fatigue curvefit to the fatigue data was −44 % for normal ligaments (Table 4, Fig. 1a) and 3 % for healing ligaments (Table 4, Fig. 2a). Using a power-law regression, the percent difference in slope (constant $-V$) comparing predicted to actual fatigue response was −29 % for normal ligaments and 7 % for healing ligaments (Table 4).

**Table 3:** Fatigue lifetime represented by the curvefit to the data as a percentage of the predicted fatigue lifetime from time-dependent damage

Model and Ligament	$T_{\mathrm{life},\mathrm{fatigue},\mathrm{data}}/T_{\mathrm{life},\mathrm{fatigue},\mathrm{predict}}$ (%) Exponential Regression	$T_{\mathrm{life},\mathrm{fatigue},\mathrm{data}}/T_{\mathrm{life},\mathrm{fatigue},\mathrm{predict}}$ (%) Power-Law Regression
**Test Stress**		
Normal Ligament		
30 %UTS–60 %UTS	11 %–3 %	7 %–3 %
15 %UTS–60 %UTS	25 %–3 %	15 %–3 %
Healing Ligament		
30 %UTS–60 %UTS	5 %–5 %	4 %–6 %
30 %UTS–80 %UTS	5 %–6 %	4 %–6 %
**Initial Strain**		
Normal Ligament		
0.042–0.114 mm/mm	45 %–0.8 %	33 %–1 %
0.042–0.118 mm/mm	45 %–0.7 %	33 %–1 %
Healing Ligament		
0.042–0.114 mm/mm	9 %–0.4 %	9 %–0.7 %
0.035–0.114 mm/mm	12 %–0.4 %	14 %–0.7 %

**Table 4:** Percent difference in slope comparing the fatigue prediction from time-dependent damage to the fatigue curvefit to the fatigue data

Model and Ligament	Percent Difference in Slope Constant −*B* Exponential Regression	Percent Difference in Slope Constant −*V* Power-Law Regression
**Test Stress**		
Normal Ligament	$ -44~\% $	$ -29~\%$
Healing Ligament	$ 3~\% $	$ 7~\%$
**Initial Strain**		
Normal Ligament	$ -62~\% $	$ -49~\%$
Healing Ligament	$ -30~\% $	$ -30~\%$

#### Initial strain

For the initial strain models, the fatigue lifetime ($T_{\mathrm{life},\mathrm{fatigue},\mathrm{data}}$) was less than the predicted fatigue lifetime ($T_{\mathrm{life},\mathrm{fatigue},\mathrm{predict}}$) for both normal and healing ligaments. Evaluating the normal and healing ligaments at similar strains (0.042–0.114 mm/mm) where fatigue lifetime represented by the curvefit to the data was expressed as a percentage of the predicted fatigue lifetime revealed similar values for normal and healing ligaments at the higher strain but greater values for normal ligaments than healing ligaments at the lower strain (Table 3). Using an exponential regression, the percent difference in slopes comparing predicted fatigue to actual fatigue responses was −62 % for normal ligaments (Table 4, Fig. 1c) and −30 % for healing ligaments (Table 4, Fig. 2c). Using a power-law regression, the percent difference in slopes was −49 % for normal ligaments and −30 % for healing ligaments (Table 4).

For healing ligaments, the highest coefficients of determination to the creep data and fatigue data were achieved using initial strain models with exponential regression (creep $r^{2} = 0.62$; Table 2 and fatigue $r^{2} = 0.78$; Fig. 2). These models were greatly affected by the data at the highest strains from the 80 %UTS tests. If that data was removed from the creep ($n=1$) and fatigue ($n=2$) exponential regressions, the coefficients of determination decreased substantially (creep $r^{2} = 0.40$ and fatigue $r^{2} = 0.30$). However, the resulting fatigue prediction still had a percent difference in slope of −30 %, and the fatigue lifetime as percent of predicted lifetime was 11 %–4 % for the strain range of 0.042–0.114 mm/mm.

### Non-interrupted creep

The majority of the creep tests that were performed were interrupted with occasional unloading/reloading cycles to monitor modulus. In only a few cases, creep tests were performed where the sustained loading was not interrupted but the MCL was loaded from $+1~\mbox{N}$ to a force corresponding to the desired test stress using a sine wave at 1 Hz and then that constant force was maintained throughout the test: 60 %UTS for normal MCLs from Burgunder rabbits ($n=3$) (Thornton et al. 2007a) and 45 %UTS for healing MCLs from New Zealand White rabbits ($n=2$) (Thornton and Bailey 2013). Lifetimes for non-interrupted creep, interrupted creep and fatigue were compared using Wilcoxon signed-rank tests for paired data and Mann–Whitney $U$ tests for unpaired data.

For normal ligaments tested at 60 %UTS, the lifetime when the creep test was not interrupted with unloading/reloading cycles was greater than the lifetime when the creep test was interrupted ($p= 0.03$; Table 5). The normal ligaments tested at 60 %UTS had greater lifetime during non-interrupted creep compared with fatigue ($p= 0.03$; Table 5). If the data for the MCLs from the New Zealand White rabbits tested at 60 %UTS (Table 1) was included with the fatigue and interrupted creep for MCLs from the Burgunder rabbits, then the lifetime for non-interrupted creep was still different than the lifetimes for interrupted creep and fatigue ($p \leq 0.02$). For healing ligaments tested at 45 %UTS, no difference in lifetime was detected when the creep test was not interrupted or interrupted with unloading/reloading cycles (Table 5). All of the lifetimes of healing ligaments tested at 45 %UTS were greater when exposed to non-interrupted creep compared with fatigue ($p = 0.06$; Table 5).

**Table 5:** Lifetimes for fatigue, interrupted creep and non-interrupted creep of normal and healing ligaments

Ligament and Test	Lifetime (s)	*n*
**Normal MCL (Burgunder)** 60 %$\mathrm{UTS}= 58.6~\mbox{MPa}$ Test Stress		
Fatigue	1.83 × 10^3^ (0.316 × 10^3^–3.65 × 10^3^)^*^^∧^	5
Interrupted Creep	21.2 × 10^3^ (10.2 × 10^3^–40.8 × 10^3^)^*^	4
Non-Interrupted Creep	86.4 × 10^3^ (58.0 × 10^3^–112 × 10^3^)	3
**Healing MCL (New Zealand White)** 45 %$\mathrm{UTS} = 8.1~\mbox{MPa}$ Test Stress		
Fatigue	21.5 × 10^3^ (0.397 × 10^3^–40.9 × 10^3^)^*^^∧^	4
Interrupted Creep	113 × 10^3^ (74.4 × 10^3^–146 × 10^3^)	4
Non-Interrupted Creep	145 × 10^3^ (131 × 10^3^–160 × 10^3^)	2

Data are shown as median (range)

Normal MCL data is from Thornton et al. (2007a), and healing MCL data is from Thornton and Bailey (2013)

^*^different than non-interrupted creep (*p* ≤ 0.06)

^∧^different than interrupted creep (*p* ≤ 0.07)

## Discussion

The results of this study revealed several important findings. Because no breed differences were detected, healing MCL data from New Zealand White rabbits were compared to normal MCL data from Burgunder and New Zealand White rabbits. Prediction of fatigue derived from the time-dependent damage from creep overestimated the fatigue lifetime for both normal and healing ligaments. At lower stress and strain, time-dependent damage was a greater relative contributor for normal ligaments than healing ligaments; however, cycle-dependent damage was a greater relative contributor with incremental increases in stress or strain for normal ligaments than healing ligaments.

The fatigue lifetime was shorter than the creep lifetime for MCLs tested at 60 %UTS for MCLs from New Zealand White rabbits. This was consistent with the observation that fatigue lifetime was shorter than creep lifetime of MCLs from Burgunder rabbits (Thornton et al. 2007a). The similarity in MCL failure, creep and fatigue responses between breeds suggests that previously published long-term creep and fatigue data, where the longest test lasted 7 days (Thornton et al. 2007a), may not have to be repeated but rather may be considered a normal rabbit MCL response regardless of breed for these two breeds.

Creep and fatigue behaviour of normal ligaments from Burgunder and New Zealand White rabbits were compared with that of healing ligaments from New Zealand White rabbits. For all models, the prediction of the fatigue derived from the time-dependent damage greatly overestimated the fatigue lifetime, which suggests that time-dependent damage alone cannot account for all of the damage accumulated during fatigue loading for either normal or healing ligaments. These findings suggest that cycle-dependent damage, in addition to time-dependent damage, has an important role in the fatigue response of ligaments. We attempted to understand the relative importance of time-dependent and cycle-dependent damage in two ways. First, we evaluated the fatigue lifetime as a percentage of the predicted lifetime from time-dependent damage where the fatigue lifetime was represented by the curvefit to the data and evaluated at similar %UTS, stress and strain for normal and healing ligaments. Second, we calculated the percent difference in the slope comparing the fatigue prediction from time-dependent damage with the curvefit to the fatigue data for normal and healing ligaments. The comparison of fatigue lifetime as a percentage of the predicted fatigue lifetime from time-dependent damage revealed different interpretations whether considering the same %UTS, stress and strain. Over similar %UTS range, the normal and healing ligaments were quite similar (Table 3). At the one comparable stress (lowest for normal ligaments but highest for healing ligaments), normal ligaments had greater values than healing ligaments. Over similar strain range, normal ligaments had greater values than healing ligaments at lower strain but similar values to healing ligaments at higher strains. Taken together, these findings suggest that time-dependent damage was a greater relative contributor for normal ligaments than healing ligaments at lower strain (or stress); however, as the strain (or stress) increased, the relative contribution of cycle-dependent damage increased. The relative importance of cycle-dependent damage with incremental increases in strain or stress was evaluated as the percent difference in slopes between the fatigue lifetime and predicted lifetime. When comparing similar models, the percent difference in slopes was consistently larger for normal ligaments than healing ligaments. The difference in the slope indicated that, for the same increase in strain (or stress), there was a greater decrease in the fatigue lifetime than that predicted from the time-dependent damage. That greater decrease was related to the cycle-dependent damage. The greater difference in slopes for the normal ligaments compared with the healing ligaments suggests that the normal ligaments are more affected by cycle-dependent damage for incremental changes in stress and strain. At lower stress and strain, time-dependent damage was a greater relative contributor for normal ligaments than healing ligaments; however, cycle-dependent damage was a greater relative contributor with incremental increases in stress or strain for normal ligaments than healing ligaments.

The 14-week healing interval for the rabbit MCL was selected because certain morphological and biochemical changes with healing have not returned to normal values. These deficiencies may contribute to the apparent difference in the relative importance of time-dependent damage and cycle-dependent damage. The ability of the healing ligament to recruit collagen fibres into load-bearing and have adequate load-sharing between the fibres may be affected due to smaller diameter collagen fibrils (Frank et al. 1992), fewer collagen crosslinks (Frank et al. 1995) and greater lubrication from increased glycosaminoglycans (Frank et al. 1983). Also, increased numbers of flaws in the matrix (Shrive et al. 1995) that create stress concentrations local to the flaws will impact the long-term loading response. While this healing occurred in vivo, the mechanical evaluation occurred in vitro. Ligaments were frozen prior to mechanical testing, which was shown previously not to affect the viscoelastic properties and failure properties of rabbit normal MCLs (Woo et al. 1986; Moon et al. 2006) and rabbit healing MCLs (Thornton and Bailey 2009). The current study did not consider the potential real-time response of live cells triggered by the mechanical response to long-term loading but rather focused on how prior in vivo healing affected the mechanical response to long-term loading by comparing the mechanical response of the ECM of healing ligaments to that of normal ligaments.

The current approach was limited to exponential or power-law regression of the creep data, assumption of constant damage rate, and introduction of oscillating stress in order to demonstrate that oscillating the time-dependent damage from creep was not sufficient to account for all of the damage accumulated during fatigue. The assumption of a constant rate of damage has been used previously to model bone (Carter and Caler 1985), tendon (Schechtman and Bader 1997; Wren et al. 2003) and ligament (Thornton and Bailey 2013). Schwab et al. (2007) developed a more complex continuum damage mechanics (CDM) model of normal ligament creep behaviour incorporating the relationship for initial rate of modulus ratio reduction and stress ratio to find a direct relationship for damage rate (Schwab et al. 2007). Additionally, that CDM model for creep incorporated a relationship between residual strength and modulus ratio, which allowed damaged fibres to potentially contribute to load-bearing through the extracellular matrix. When the fatigue was predicted as the time-dependent damage from creep using the Schwab et al. (2007) model, the fatigue lifetime was also overestimated. This suggests that modifications of expressions for damage rate and residual strength of the creep response may still not be sufficient to account for all the damage accumulated during fatigue; thus, cycle-dependent damage is an important contributor to the fatigue response.

Further evidence of the difference in the relative importance of cycle-dependent damage for normal ligaments compared to healing ligaments was found in the comparison of non-interrupted creep to interrupted creep. For the healing ligaments, the creep tests were interrupted after 3000 cycles (0.8 hours) and every 1.16 hours thereafter. For the normal ligaments, the creep tests were interrupted 21 times during the allocated test time of 24 hours or 24 hour intervals thereafter. For normal MCLs tested at 60 %UTS, the average interruption interval, after the first interruption at 3000 cycles (0.8 hours), was 1.16 hours. For normal MCLs tested at 30 %UTS and 15 %UTS, the average interruption interval, after the first interruption at 3000 cycles (0.8 hours), was 3.6 to 6 hours for 30 %UTS and 3.6 to 8.4 hours for 15 %UTS. The interrupted creep tests at the lower stresses for normal ligament had less frequent interruptions than at 60 %UTS, which suggests that the effect of interruption cycles would be less at the lower stresses. Occasional interruptions of the sustained loading of the creep test with unloading/reloading cycles to monitor modulus resulted in a decrease in the lifetime of normal ligaments tested at 60 %UTS revealed when comparing non-interrupted creep lifetime to interrupted creep lifetime (Table 5). However, occasional interruptions of the creep test did not have a detectable effect on the lifetime of healing ligaments tested at 45 %UTS. At the different test stresses examined, normal ligaments appear to be more affected by cycle-dependent damage than healing ligaments. Future studies could investigate the effect of occasional interruptions in the otherwise constant loading of a creep test by considering different test stresses and whether the apparent effect in normal ligaments and apparent lack of effect in healing ligaments would occur at similar %UTS, stress or strain.

The data from Table 5 was further analyzed by adding the ratio of interrupted creep lifetime to non-interrupted creep lifetime and the ratio of interrupted creep cycles to rupture to fatigue cycles to rupture in order to compare the result to the linear rule where the sum would be equal to 1 (Bowman and Barker 1986; Lemaitre and Desmorat 2005). The sum was 0.3 for the normal ligaments at 60 %UTS and 0.8 for the healing ligaments at 45 %UTS, which indicated that a linear rule was not supported and suggested that creep and fatigue interact. This adds to the complexity of what could account for the difference between the time-dependent damage as predicted from creep and the actual fatigue behaviour because not only should time-dependent damage and cycle-dependent damage be considered but also creep-fatigue interaction.

In summary, prediction of the fatigue derived from the time-dependent damage from creep overestimated the fatigue lifetime for both normal and healing ligaments. Healing ligament ECM has morphological and biochemical differences that contribute to mechanical differences. Lifetimes were shorter for healing ligaments compared to normal ligaments, and the fatigue lifetimes were shorter than the creep lifetimes for both normal and healing ligaments. At lower stress and strain, time-dependent damage was a greater relative contributor for normal ligaments than healing ligaments; however, cycle-dependent damage was a greater relative contributor with incremental increases in stress or strain for normal ligaments than healing ligaments. For the ECM of normal ligaments, cycle-dependent damage has a greater contribution with increases in stress or strain, which may be important with the increased loading on a normal ligament when a complementary ligament restraint is injured. For the ECM of healing ligaments when the ligament itself is injured, the consistent contribution of cycle-dependent damage over a range of stresses and strain also has important implications when designing rehabilitation exercises that result in sustained and repeated loading, suggesting that repeated loading permits more damage to accumulate than sustained loading.
